# ChloroSSRdb: a repository of perfect and imperfect chloroplastic simple sequence repeats (cpSSRs) of green plants

**DOI:** 10.1093/database/bau107

**Published:** 2014-11-06

**Authors:** Aditi Kapil, Piyush Kant Rai, Asheesh Shanker

**Affiliations:** ^1^Department of Bioinformatics, Banasthali University, Banasthali, Rajasthan 304022, India and ^2^Department of Mathematics and Statistics, Banasthali University, Banasthali, Rajasthan 304022, India

## Abstract

Simple sequence repeats (SSRs) are regions in DNA sequence that contain repeating motifs of length 1–6 nucleotides. These repeats are ubiquitously present and are found in both coding and non-coding regions of genome. A total of 534 complete chloroplast genome sequences (as on 18 September 2014) of Viridiplantae are available at NCBI organelle genome resource. It provides opportunity to mine these genomes for the detection of SSRs and store them in the form of a database. In an attempt to properly manage and retrieve chloroplastic SSRs, we designed ChloroSSRdb which is a relational database developed using SQL server 2008 and accessed through ASP.NET. It provides information of all the three types (perfect, imperfect and compound) of SSRs. At present, ChloroSSRdb contains 124 430 mined SSRs, with majority lying in non-coding region. Out of these, PCR primers were designed for 118 249 SSRs. Tetranucleotide repeats (47 079) were found to be the most frequent repeat type, whereas hexanucleotide repeats (6414) being the least abundant. Additionally, in each species statistical analyses were performed to calculate relative frequency, correlation coefficient and chi-square statistics of perfect and imperfect SSRs. In accordance with the growing interest in SSR studies, ChloroSSRdb will prove to be a useful resource in developing genetic markers, phylogenetic analysis, genetic mapping, etc. Moreover, it will serve as a ready reference for mined SSRs in available chloroplast genomes of green plants.

**Database URL:** www.compubio.in/chlorossrdb/

## Introduction

Chloroplasts are semiautonomous organelles having their own genome (1) and considered to be derived from cyanobacteria through endosymbiosis (2). Apart from their well-known function of photosynthesis, i.e. the conversion of light energy to chemical energy, chloroplasts are known to play a role in the synthesis of starch, fatty acids, pigments and amino acids (3). Moreover, chloroplast genome sequences have also been widely used in plant systematics (4–7) and simple sequence repeats (SSRs) mining (8, 9).

SSRs also known as microsatellites are the specific portions of DNA sequence that contain clusters of tandem repeating motifs of length 1–6 nucleotides (10). These repeats are supposed to be generated by slippage during replication (11) and are present in both coding as well as non-coding regions of DNA. These repeats show less polymorphism in coding sequences as compared to non-coding sequences (12). The specificity, reproducibility, co-dominance and hypervariability of SSRs make them potential molecular markers (13). The conserved flanking sequences of SSRs help in the designing of PCR primers which can be further used for the amplification of repeat sequence (14). SSRs can be used for genotyping and population level evolutionary studies (15). Moreover, these repeats play an important role in gene regulation and the importance of SSRs in the evolution of coding and non-coding regions has been proved (16, 17). Chloroplastic SSRs (cpSSRs) also play an important role in population genetics and evolutionary studies of plants (18).

With the increase in availability of expressed sequence tags (ESTs) and complete genome sequences in biological databases, *in silico* mining approaches proved to be useful in the identification of SSRs (8, 9, 19, 20). Consequently, a large number of SSR-specific databases including MICdb (21), Cotton Marker Database (22), EuMicroSatdb (23), PIPEMicroDB (24), ChloroMitoSSRDB (25) and MitoSatPlant (26) have been developed.

This study is an attempt to develop a comprehensive, user friendly, specialized database of cpSSRs mined from complete chloroplast genome sequences of green plants (Viridiplantae). To the best of our knowledge, among all the SSR-specific databases available, this is the only database of SSRs which provides information about perfect, imperfect and compound SSRs along with statistical analyses of the repeats identified. The database includes pre-calculated density of SSRs, average length of SSRs, repeat type frequencies, chi-square statistics, relative values, their correlation coefficient, SSR-specific PCR primers, etc.

## Materials and Methods

### Data mining and statistical analysis

**Figure 1.**
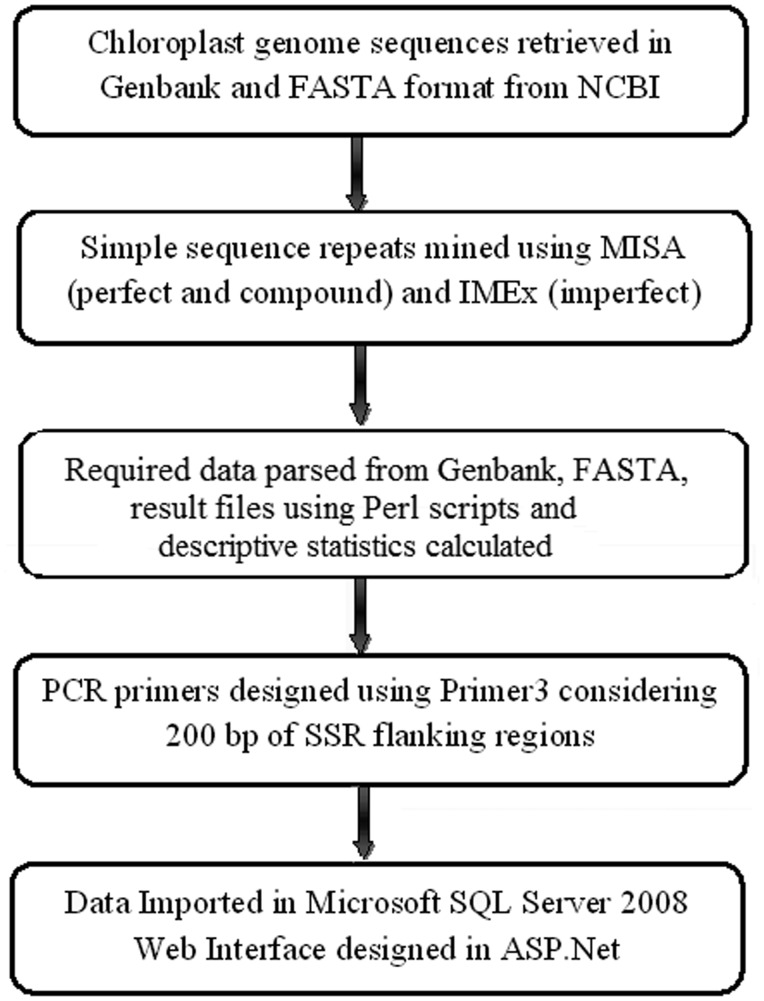
The workflow of ChloroSSRdb.

The information included in the ChloroSSRdb was mined from 534 completely sequenced chloroplast genomes of Viridiplantae. These genomes belong to 39 algae, 9 bryophytes, 17 pteridophytes, 41 gymnosperms and 428 angiosperms. Genbank (*.gbk) and fasta (*.fna) formatted files of these genomes were retrieved from NCBI (ftp://ftp.ncbi.nih.gov/genomes/Chloroplasts/plastids/). For SSRs scanning, a minimum length criterion of repeating motif ≥12 mono, ≥6 di, ≥4 tri and ≥3 for tetra, penta and hexa nucleotide repeats was used. Perfect and compound SSRs were identified using microsatellite identification tool (MISA; http://pgrc.ipk-gatersleben.de/misa/down load/misa. pl) which fetches non-redundant perfect and compound microsatellites from a given DNA sequence. The number of intervening nucleotides between motifs of compound microsatellites was set to 0. Imperfect Microsatellite Extractor (IMEx 2.0) (27) with the imperfection percentage of 10% was employed to get imperfect SSRs. In-house developed Perl scripts were used to parse results of MISA and IMEx along with additional information from Genbank and fasta formatted files. Primer3 (http://bioinfo.ut.ee/primer3-0.4.0/) (28) with default parameters was used to generate PCR primers considering 200 bases of SSR flanking regions. In addition to this, chi-square (χ^2^) values of identified SSRs, their relative values and correlation coefficient (*r*) between the relative values of perfect and imperfect SSRs were calculated as follows:

$$\chi^{2}=\sum_{i=1}^{n}\frac{{\left(O_{i}-E_{i}\right)}^{2}}{E_{i}},$$

where *O_i_* and *E_i_* are the observed and expected values of i^th^ observation.

$$r=\frac{1/n\sum_{i=1}^{n}\left(X_{i}-\bar{X}\right)\left(Y_{i}-\bar{Y}\right)}{\sqrt{1/n\sum_{i=1}^{n}{\left(X_{i}-\bar{X}\right)}^{2}}\sqrt{1/n\sum_{i=1}^{n}{\left(Y_{i}-\bar{Y}\right)}^{2}}},$$

where *X* and *Y* are the relative frequencies of perfect and imperfect SSRs, respectively. The workflow of ChloroSSRdb is presented in Figure 1.

### Database design and web interface

ChloroSSRdb is based on relational database management system and was developed using SQL server 2008. It follows client–server architecture in which the communication is one-to-one and takes place between client and server without any intermediate. The database contains a total of 19 tables. Each table uses the accession number of chloroplast genome sequence as a unique identifier (primary key). The database can be accessed through an interactive, easy to use interface developed in ASP.NET.

## Results and Discussion

The front end of ChloroSSRdb provides a user-friendly browsing facility to look for the SSRs information in respective organism. Navigation to different pages are provided to link mined data with other information and every page contains a hyperlink to download the displayed information in MS Excel file.

**Figure 2.**
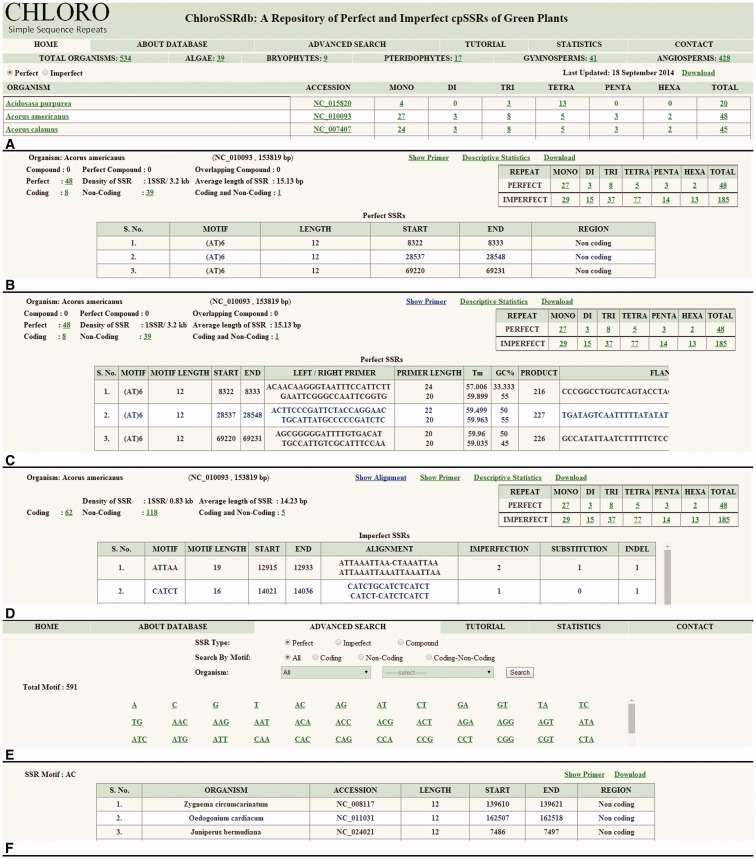
Browsing activity of ChloroSSRdb. (**A**) Home page showing name of organisms with SSR (mono–hexa) frequency. (**B**) Information of selected organism. (**C**) Primer sequences of SSRs. (**D**) Alignment of imperfect SSR with expected perfect SSR. (**E**) Advanced search page. (**F**) Results of advanced search.

The home page (Figure 2A) displays a broader classification of chloroplast genomes as algae, bryophytes, pteridophytes, gymnosperms and angiosperms where angiosperms are further classified as monocots and eudicots. The mined information of mono–hexa repeat types can be accessed for both perfect and imperfect SSRs (Figure 2A). The organism name is directly linked to the taxonomic page and its accession id to GenBank page at NCBI which enable user to fetch taxonomic and genomic DNA information, respectively. The repeat counts are hotlinked to the information page where frequency of mono–hexa repeats, perfect, imperfect, compound, overlapping compound [overlap of few bases of previous SSR with next SSR, e.g. (ACC)_n_(CT)_n_] and perfect compound SSRs are displayed. Additional information such as coding (CDS, tRNA, rRNA), non-coding (intergenic and intragenic), coding–non-coding regions (occurrence of few bases in coding as well as in non-coding regions or vice versa), gene id, protein id, total density and average length of SSRs are provided (Figure 2B). The genomic location of SSRs provided with additional information will facilitate in the determination of their functional roles. Furthermore, links to designed PCR primers and alignment (to report substitutions or indels in imperfect SSRs) are available (Figure 2C and D). SSR flanking regions of 200 nucleotides are available with primer sequences. These primers can be used to develop SSR-based markers, for transferability studies across species, within a genus, across genera, and for the experimental validation of polymorphism. In addition to this, the designed primers can be used to check length polymorphism of SSRs in different species which can be helpful in species identification.

**Figure 3.**
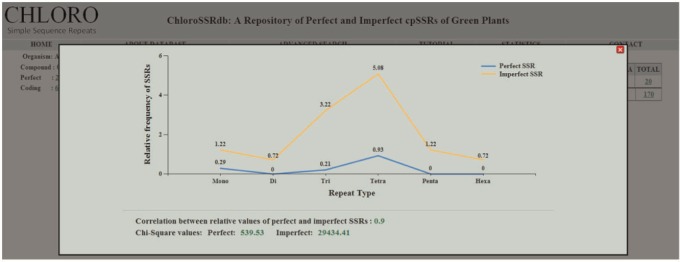
Chi-square statistic, relative values of perfect and imperfect SSRs along with their correlation coefficient.

The advanced search option caters information on SSRs by motif specific search and filters the results on the basis of region, repeat type and the organism classification (Figure 2E and F). The descriptive statistics link (Figure 2B–D) opens a graphical representation (Figure 3) which depicts the relative frequencies (mono–hexa) of perfect and imperfect SSRs, their correlation coefficient value and the chi-square statistics. The range of correlation coefficients are (−0.33, 0.98) where 50% of the species have significantly positive value. Moreover, some of the cases are having significantly negative correlation coefficient values. The calculated chi-square values in almost all cases are much greater than the tabulated value at 5% level of significance (5 df). This clearly rejects the null hypothesis, i.e. the data follow a natural specified distribution, which concludes that the observed values are significant and the differences are not only due to chance.

For user’s ease, a tutorial is provided to easily browse ChloroSSRdb. The statistics tab displays overall statistical information of ChloroSSRdb which includes largest and smallest genomes mined, total number of SSRs mined, number of primers designed, most and least abundant repeats and organism with maximum SSR density. Additionally, a bar graph is provided that depicts the year-wise submission of chloroplast genomes of green plants in NCBI.

## Conclusion

An easy to use, comprehensive database of cpSSRs mined from 534 chloroplast genomes has been developed. We hope that ChloroSSRdb will appear to be a useful resource for researchers interested in the study of cpSSRs. The statistical analyses of mined data will aid the scientific community to understand the distribution and pattern of cpSSRs evolution among plant lineages. ChloroSSRdb will be regularly updated in accordance with the sequence entries in NCBI and we will expand it by providing as much information as possible.
